# The First Online Capillary Electrophoresis-Microscale Thermophoresis (CE-MST) Method for the Analysis of Dynamic Equilibria—The Determination of the Acidity Constant of Fluorescein Isothiocyanate

**DOI:** 10.3390/molecules27155010

**Published:** 2022-08-06

**Authors:** Paweł Mateusz Nowak, Maria Klag, Gabriela Kózka, Małgorzata Gołąb, Michał Woźniakiewicz

**Affiliations:** Department of Analytical Chemistry, Faculty of Chemistry, Jagiellonian University, Gronostajowa 2, 30-387 Kraków, Poland

**Keywords:** acid-base equilibrium, capillary electrophoresis, microscale thermophoresis, molecular interactions, fluorescence

## Abstract

This article presents the first successful application of a capillary electrophoresis-microscale thermophoresis tandem technique (CE-MST) for determining the values of equilibrium constant, realized by connecting online the CE and MST instruments using a fused-silica capillary. The acid-base dissociation of fluorescein isothiocyanate, expressed by the acidity constant value (p*K*_a_), was used as a model. The measurement procedure consisted of introducing a mixture containing the analyte and a deliberately added interferent into the CE capillary, electrophoretic separation of the analyte from the interferent, the detection of the analyte with a CE-integrated detector, detection with a MST detector, and then stopping the flow temporarily by turning off the voltage source to conduct the thermophoretic measurement. The analysis of migration times, peak areas and MST responses obtained concurrently for the same sample allowed us to determine the p*K*_a_ value using three independent methods integrated within one instrumentation. The analyte was effectively separated from the interferent, and the acidity values turned out to be consistent with each other. An attempt was also made to replace the standard commercial CE instrument with a home-made portable CE setup. As a result, the similar p*K*_a_ value was obtained, at the same time proving the possibility of increasing cost efficiency and reducing energy consumption. Overall, the CE-MST technique has a number of limitations, but its unique analytical capabilities may be beneficial for some applications, especially when sample separation is needed prior to the thermophoretic measurement.

## 1. Introduction

The microscale thermophoresis technique (MST) is a modern and often used tool for studying dynamic equilibria and molecular processes in a solution, including the analysis of ligand–target affinity, stability, aggregation, conformational changes, acid-base dissociation, thermodynamics of these processes as well as kinetics of enzymatic reactions [1,2,3,4,5,6,7,8]. It analyzes the change in the fluorescence signal coming from a previously labeled or natural fluorophore, detected within a small area of the sample heated with an infrared laser. In the most common configuration, the sample is manually introduced into glass capillary by inserting it directly into sample solution. The filled capillaries are afterwards placed on a special tray inside the MST instrument. As a result of creating a temperature gradient of the order of 5–10 K, two phenomena occur: temperature-related intensity change (TRIC) and thermophoresis, i.e., thermal diffusion or the Soret effect [3,9]. The simultaneous occurrence of both phenomena is usually seen as a decrease in fluorescence, which gradually weakens over time, reaching the equilibrium state after about half a minute. The resulting plot of the change in fluorescence over time, called the MST response, can be described by the parameter “normalized fluorescence”, see Equation (1):

(1) $$F_{\mathrm{norm}}=\frac{F_{\mathrm{hot}}}{F_{\mathrm{cold}}}$$

where F_norm_ is the normalized fluorescence, F_hot_ is the intensity of fluorescence measured at a selected time point after forming the microscopic temperature gradient by an IR laser and F_cold_ is the intensity of fluorescence measured before heating.

The value of this parameter depends on many components such as charge, particle size, conformation and structure of the solvation shell [9]. It carries information about the occurrence of molecular interactions; therefore, the MST technique can be used to determine the values of equilibrium constants and to track these processes. The undoubted advantages of MST are: a unique physicochemical basis and a wide spectrum of phenomena that can be studied, real-time analysis of additional processes such as aggregation and photobleaching, no need for immobilization, analysis directly in a solution that may be similar in composition to physiological conditions, a wide range of molecules from ions to biopolymers, high sensitivity, possibility of thermodynamic investigations by performing measurements in different precisely controlled temperatures, low sample consumption, short analysis time and ease of use combined with intuitive software. Nonetheless, the biggest disadvantages are the need to use fluorescence as a detection system, the relatively high cost of the MST device and consumables and the need to use samples of known composition, free from potential interferents.

The capillary electrophoresis (CE) technique may be considered an alternative and to some extent a similar technique to MST, since both utilize capillaries and the movement of molecules in the physicochemical gradient. Their specifics are nevertheless evidently different. CE is a separation technique that enables the analysis of equilibrium interactions and processes simultaneously for many analytes, after their prior separation and purification against potential interferents. It also enables the use of various types of detectors-UV, UV-VIS, fluorescence, mass spectrometers (MS) and others, and can be implemented with the use of advanced CE instruments, as well as simple and much cheaper home-made and portable setups and chips. However, the use of CE to analyze some types of interactions can be problematic or even impossible. One of the main problems may be insensitivity to certain processes, weaker reproducibility, a tedious process of preparing the instrument and experiment as well as the inability to effectively separate some mixtures in parallel with the analysis of occurring interactions [10,11,12,13,14,15,16,17].

A few years ago, our research group proposed the original concept of combining CE and MST into one tandem technique in an online flow format (CE-MST) [18]. This possibility was confirmed by conducting some preliminary measurements in this unconventional setup. The basis was the direct connection of the CE and MST instruments by means of a single, flexible, and long CE-compatible fused silica capillary, led outside the CE device (similarly to the connection of CE with MS detector). The outer part of the CE capillary was inserted into the MST instrument, placed directly inside the wider MST glass capillary and immobilized on a standard tray used in the MST setup. The distal capillary part was again led outside and placed in a vial into which a ground electrode was inserted to close the electrical circuit. The fundamental principle of measurement consisted of performing an electrophoretic separation upon the application of high voltage to the ends of the capillary and stopping the flow when the analyte-containing segment reached the MST detector, which was termed the “stop-flow mode”. At this point, the thermophoretic measurement took place in the same way as in the case of the regular MST method. After collecting the signal (MST response), the voltage was reapplied and the procedure could be repeated for the next segment reaching the MST detector containing a different analyte separated from the previous one as a result of the electrophoresis process. More details on the methodology of this experiment are available in the original article [18].

The main assumption of the CE-MST technique was to increase the analytical potential of MST by taking advantage of performing electrophoretic separation immediately before thermophoretic measurement in an online flow system. The main added value was the possibility of separating the analyte from interferents (purification), simultaneous analysis of several analytes separated from each other, e.g., isomers, and simultaneous collection of electrophoretic (electropherogram) and thermophoretic (MST response) data that could be complementary. In an ideal scenario, a given molecular process could be studied in one experiment using two orthogonal techniques, which would result in the collection of extended information, increasing the reliability of the obtained results. In principle, this is supposed to take place at the expense of some obvious losses associated with the use of the CE-MST interface: a decrease in sensitivity (the internal diameter of the CE capillary is several times smaller than the MST capillary), increased cost and time consumption as well as the elevated degree of its overall complexity. The aforementioned preliminary studies showed a promising prospect for the development of this technique [18], demonstrating that from a technical point of view, such a combination is feasible. Nevertheless, the CE-MST technique has not been applied so far to analyze any type of interaction and equilibrium, thus the question of its actual usefulness and feasibility remains open.

This article presents the first ever application of the tandem CE-MST system to the analysis of an equilibrium process. It was primarily aimed at finding out whether the expected benefits provided by the integration of both techniques can actually be achieved. As a model equilibrium process to be studied, the acid-base dissociation characterized by the value of the acidity constant (p*K*_a_) was selected. Such a method using the standard MST approach was developed by our group and recently published in the *Molecules* journal [19], and it seemed to be an ideal candidate to be transferred into the CE-MST system due to its general simplicity. The integration with the CE technique was supposed to enable sample separation from potential interferents and the simultaneous application of the electrophoretic and fluorimetric methods to study the acidity [20].

## 2. Results and Discussion

### 2.1. Preliminary Study

#### 2.1.1. Sensitivity in the CE and MST Capillaries

The decrease in sensitivity was one of the main expected side effects associated with the use of the CE-MST interface realized by means of a common CE capillary. The recognition of this effect was crucial for the development of an effective method of p*K*_a_ determination using CE-MST. For this purpose, preliminary tests were carried out in which the fluorescence intensities recorded by the MST detector were compared for the MST and CE capillaries, without heating the sample (IR laser turned off), in the entire tested pH range. A simplified system was used for this provisional study. The CE capillary, upon burning off the detection window, was placed inside the MST capillary, analogously to that in the target CE-MST interface. The simplification was that its inlet part was not installed in the CE instrument, but was kept outside, similar to its outlet part. The sample was manually introduced into the CE capillary, using the vacuum generated by the syringe with a needle which was connected to the capillary with a sealed tube (rubber connector). This was shown in Figures S1–S3 in the Supplementary Material (SM). The results obtained with this arrangement are shown in Table 1 and Table S1 in the SM.

The presented data show that, as expected, the sensitivity of the measurement in the CE capillary is significantly lower in relation to the routinely used MST capillary. Obtaining similar values of the fluorescence intensity at the same excitation light power (LED = 10%) required the use of a 20-fold higher concentration of FITC. Quantitatively, the decrease in sensitivity at a given pH is illustrated by the normalized signal ratio (NSR) values (the value obtained for the MST capillary with LED set up at 10% power divided by the values obtained for the CE capillary and the same LED power, normalized with respect to the difference in FITC concentrations), see Equation (2):

(2) $$\mathrm{NSR}=\frac{\mathrm{MS}T_{\mathrm{LED}10\%}}{CE_{\mathrm{LED}10\%}}\cdot 20$$

where MST_LED10%_ is the signal measured in the MST capillary with LED = 10% and CE_LED10%_ is the signal measured in the CE capillary with the same LED setting.

The NSR values range from about 5 to 30, showing a strong pH dependence. Similar values were recorded for both electrolytes that differed in ionic composition (comparison between Table 1 for phosphate-based electrolytes and Table S1 for acetate-based electrolytes in SM).

These results clearly show the real extent of sensitivity deterioration that should be taken into account in the case of the CE-MST technique. The observed dependence of NSR on pH can be explained by the effect of background fluorescence. It decreases with pH more appreciably for the MST capillary, for which the sensitivity is higher. In general, it can be concluded that the observed difference in sensitivity is not drastic and can be relatively easily compensated by an increase in analyte concentration, in this case about 20 times, and thus unequivocally does not exclude the possibility of analyzing the acidity of FITC by means of CE-MST.

It is also worth paying attention to the possibility of improving the sensitivity of the method by increasing the intensity of the excitation light, as evidenced by the values measured at different LED settings in the MST capillary. However, it should be highlighted that this effect is not linear. As can be seen from the data in Table 1, the change in LED from 2 to 10% (5 times) resulted in, on average, a 14 times increase in signal, while the change from 10 to 40% (4 times) only resulted in a 4.5 times increase. Accordingly, a better effect can be obtained by manipulating the LED setting in the low value range, which may help to avoid the negative consequences associated with the use of high light powers, such as the photobleaching effect.

Apart from compensating for the decrease in sensitivity by increasing the concentration and intensity of the excitation light, there are other potential possibilities. These are primarily the use of CE capillaries with a larger internal diameter and the use of known techniques for preconcentrating the analyte directly in the CE capillary after its injection [21,22,23]. Although increasing the internal diameter from the 75 µm used in this experiment to 100 or even 200 µm should not cause significant problems, electrophoretic methods of concentrating the analyte in the capillary, for example by its stacking, would increase the level of complexity of the entire measurement methodology.

#### 2.1.2. MST Responses in the CE and MST Capillaries

**Figure 1:**
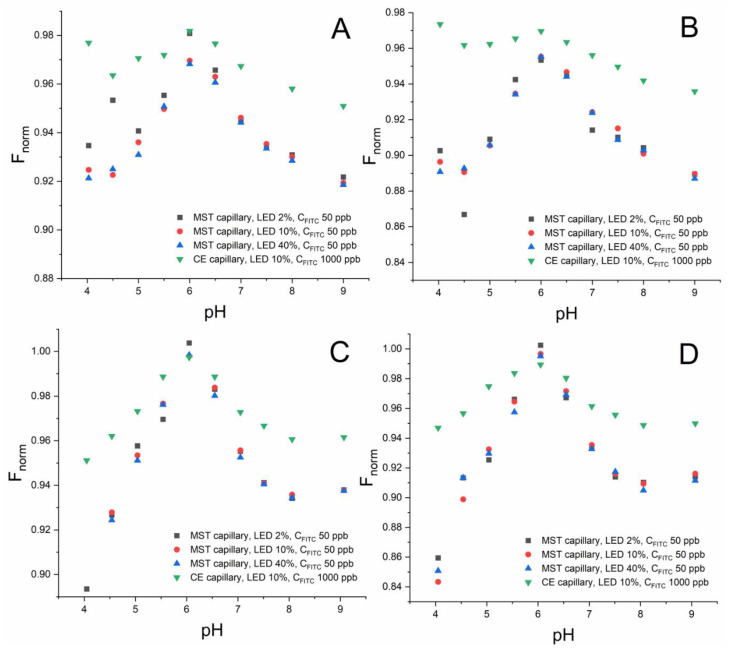
Dependence of the MST response (F_norm_) on pH obtained using the MST and CE capillaries; (**A**) phosphate-based electrolytes, 5 s of heating; (**B**) phosphate-based electrolytes, 30 s of heating; (**C**) acetate-based electrolytes, 5 s of heating; (**D**) acetate-based electrolytes, 30 s of heating; LED—relative power of light emitting diode (intensity of excitation light set up with the MST device).

Subsequently, the dependence of the F_norm_ parameter on pH obtained using the same simplified instrumental system was analyzed. The F_norm_ values were calculated for the F_hot_ values read at two different times, 5 and 30 s from turning on the IR laser (Equation (1)). The complete set of data is presented in Figure 1.

As can be seen from Figure 1, in each case, the relationship obtained for the MST and CE capillaries differs significantly in terms of the absolute values of F_norm_, but it appears very similar in terms of a dependance on pH. In particular, the pH range of 6–8 should be taken into account, which is used directly for the determination of p*K*_a_ related to the phenol group of FITC by the thermophoretic technique [19]. Regardless of the ionic composition and the heating time of the sample after which the F_hot_ reading was taken, the obtained relationships between the phenomena of thermophoresis/TRIC and pH remain in full agreement for both capillaries. This suggests that the p*K*_a_ values determined using the target CE-MST coupled system should be consistent with the classical MST method. Moreover, the selection of the excitation light power also does not seem to have a significant impact, which may be important during possible attempts to compensate for the decrease in sensitivity with the LED setting. The minor differences in F_norm_ observed for the particular pH values for the phosphate- and acetate-based electrolytes stem probably from the dependence of thermophoresis and TRIC on ionic composition. They seem to have no direct impact on p*K*_a_ value.

Regarding the pH range below 6, the data obtained differ more significantly. The observed dependence on pH in this range is probably, as it was stated in our previous work [19], due to the ionization of another group (carboxyl), which entails the opposite effect, i.e., an increase in F_norm_ with pH. The fluorescence intensity, and hence the sensitivity, is however low (Table 1 and Table S1); thus, the usefulness of this pH range for the determination of the carboxyl group’s p*K*_a_ is doubtful.

In addition, the differences in the F_norm_ values between the MST and CE capillaries at given pH values allow us to make a hypothesis that the temperature gradient generated in the CE capillary is significantly lower, and thus the phenomena of thermophoresis and TRIC are weakened. Approximately, taking into account the scale of the F_norm_ change with pH, this effect is about two times. This appears to be justified in the absorption of IR radiation by the walls of both MST and CE capillaries, used simultaneously “one-inside-the other”. However, this effect should not considerably affect the obtained p*K*_a_ values, but only diminish a minimal concentration of FITC at which it is possible to determine p*K*_a_.

#### 2.1.3. Repeatability of MST Responses in the CE and MST Capillaries

The last objective of the preliminary research was to verify the repeatability of the analysis. It was carried out based on the relative standard deviation (RSD) values, obtained for the F_norm_ from the three independent, consecutive measurements. The complete data related to both electrolyte types, two different F_hot_ reading times and the entire pH range are provided in Tables S2–S5 in the SM. It is evident that in each case, the RSD values are low and only in a small number of cases do they exceed 1%. Importantly, in the case of the CE capillary, the mean RSD values are not higher, and are often even lower, than in the case of the MST capillary. This proves a satisfactory repeatability of the method both in the variant with the traditional MST capillary and in the non-standard variant with the CE capillary. Interestingly, the repeatability did not deteriorate despite the rather significant decrease in sensitivity and the probable change in conditions within the MST detector site, due to the placement of an additional object (second capillary).

### 2.2. Full Integration of the CE and MST Instruments

**Figure 2:**
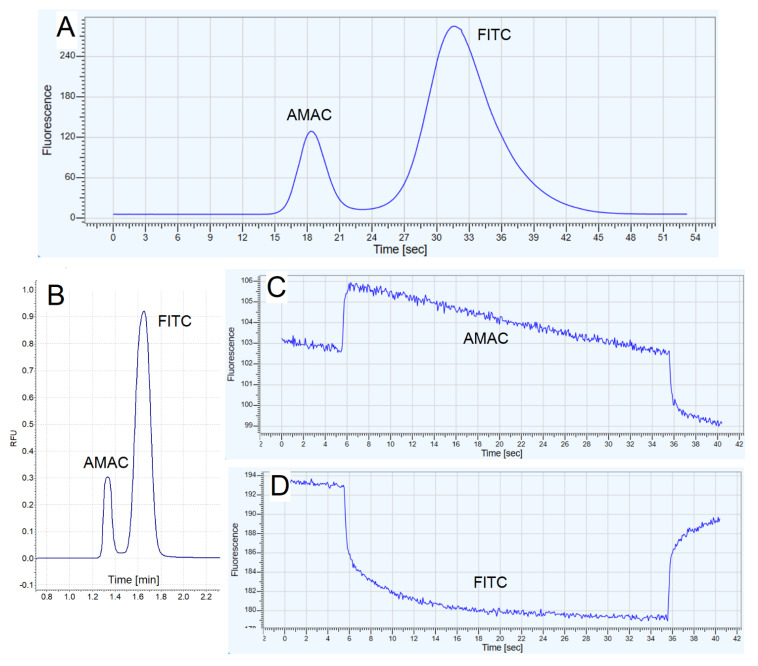
The examples of electrophoretic and thermophoretic data obtained for the analysis of AMAC–FITC mixture at pH close to 7.0 in the phosphate buffer: (**A**) electropherogram recorded with the MST detector showing the separation of AMAC and FITC, without stopping the flow and without performing the thermophoretic measurement, signal registration was started manually based on the pre-estimated migration times; (**B**) electropherogram obtained with the LIF detector integrated with the CE instrument; (**C**) MST signal obtained for the AMAC by stopping the flow when AMAC-containing zone reached the MST detector; (**D**) MST signal obtained for the FITC by stopping the flow when FITC-containing zone reached the MST detector. Fluorescence intensity is shown in relative units.

The presented results of the preliminary studies allowed us to assume that the determination of the p*K*_a_ value using the fully integrated CE-MST system will be possible with satisfactory precision and accuracy, despite a certain decrease in sensitivity, which can be compensated for by an approximately 20-fold increase in the analyte concentration. To verify this hypothesis, the CE and MST instruments were connected as described in Section 3.4 (also see Figures S4–S7 in SM for visualization), and then a series of measurements were carried out consisting of the automatic injection of the sample containing the analyte (FITC) and an intentionally added interferent (AMAC) into the CE capillary, its electrophoretic separation, transport of the separated FITC segment through the LIF detector to the MST instrument, stopping the flow by temporarily switching off the voltage source and recording MST responses to determine the F_norm_ value. Alternatively, the flow was stopped to record the MST response for AMAC instead of FITC, or its stopping was not conducted in order to obtain a complete electropherogram with the MST fluorescence detector, as shown in Figure 2. To reduce analysis time, the additional pressure of 13.8 kPa (2.0 psi) was applied to assist electrokinetic migration (the separation voltage was 30 kV). We decided to use AMAC because it is, apart from FITC, the main native fluorophore used in our laboratory. 

In Figure 2A,B, it can be seen that the sample was successfully separated and the electropherograms obtained with both detectors located in-line are similar. The resolution was not deteriorated between two detection sides (LIF and MST detectors). An interesting observation is that the MST responses recorded for AMAC (Figure 2C) and FITC (Figure 2D) are opposite, i.e., as far as FITC displays a commonly observed decrease in fluorescence in the whole time range, AMAC displays a drastic increase in the signal. It confirms that the analytes separated with the CE-MST can be distinguished directly from the shape of MST responses and it can be used as an additional criterion of the qualitative analysis in the case of sample of unknown composition. 

**Figure 3:**
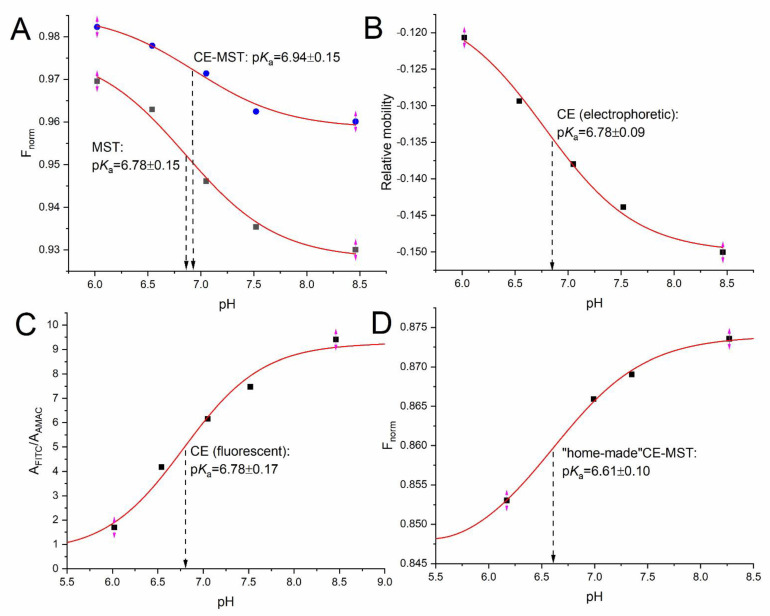
Non-linear models and acidity constant values obtained for: (**A**) the standard MST method versus its online coupling with CE (CE-MST); (**B**) electrophoretic method based on electrophoretic mobilities; (**C**) fluorescent method based on the relative signal intensities recorded by LIF detector; and (**D**) the prototype CE-MST system realized in a simplified and portable format. Excitation light power was the same for both MST and CE-MST setups (10%), F_hot_ was read after 5 s of heating. FITC concentrations were 50 ng∙mL^−1^ (50 ppb) for MST and or 1 µg∙mL^−1^ (1000 ppb) for CE MST.

The F_norm_ values obtained for FITC in the pH range above 6 in the phosphate buffer were compared to the data obtained by using the traditional MST format, without coupling with CE. Then, the model curves were fitted to determine p*K*_a_ values, as presented in Figure 3A.

According to the assumption, it has turned out to be possible to determine reliable p*K*_a_ values using the CE-MST technique. The values obtained using the MST and CE-MST approaches are consistent; they fall within the range of the determined model fit error. Moreover, a good agreement was observed between thermophoretic and independent reference techniques, based on electrophoretic mobilities and fluorescence intensity (compare Figure 3A–C). It should be admitted that the total uncertainty of the experimentally determined p*K*_a_ values also includes the uncertainty of pH, ionic strength and temperature [24,25]. Therefore, in our opinion, even differences between the p*K*_a_ values of 0.2–0.3 units should still be considered satisfactory. In this case, compliance is better; moreover, the slightly lower p*K*_a_ value observed for the standard MST variant may be due to a slightly higher temperature gradient (Section 2.1.2), because the p*K*_a_ values for FITC decrease with increasing temperature [19].

### 2.3. Integration of the Low-Cost and Green CE Setup with MST 

Finally, an attempt was made to apply a portable home-made CE system, built up previously in our laboratory [26], and integrate it with the MST device (see Section 3.5). The visualization of this interface is shown in the SM (Figure S8). The preliminary results obtained with this prototype setup allowed us to fit the model curve and estimate p*K*_a_, shown in Figure 3D. The model was only fitted to four points since the measurements in one buffer failed, and the FITC zone was not detected with the MST. Noticeably, the opposite increasing trend was observed for the F_norm_ values against pH (compare with Figure 3A), which most probably stems from the different properties of the electrolytes used in the portable CE format due to the use of a C^4^D detector. It clearly points out how difficult is to properly predict the thermophoretic behavior of molecules in varied media. The obtained p*K*_a_ value was a little lower than in the other cases; nevertheless, taking into account the lack of temperature control leading to considerable generation of Joule heat in this system, and the previously documented decrease in p*K*_a_ with temperature [19,24], such an outcome seems to be understandable and satisfactory. The impact of lower ionic strength of electrolytes used in this system can be assumed as rather insignificant in comparison to thermal effects. 

Therefore, in general, these preliminary results look promising and prompt us to be optimistic about the analytical capabilities of the “home-made” CE-MST coupling, despite the significant shortcomings that need to be studied and addressed. It will be the purpose of our future research. Our current intention was to only show that the replacement of an expensive and highly energy-consuming CE instrument with a low-cost, greener and more portable alternative is achievable. 

## 3. Materials and Methods

### 3.1. Materials and Reagents

Fluorescein isothiocyanate (FITC) used as analyte was supplied by Sigma-Aldrich (St. Louis, MO, USA). It was dissolved in an aqueous electrolyte of a selected pH at a final concentration of 50 ng∙mL^−1^ (50 ppb) or 1 µg∙mL^−1^ (1 ppm). In the MST method, it was manually introduced into the MST capillaries by immersing them in the solution. In measurements conducted with the CE-MST system, 2-aminoacridone (AMAC) was used as intentionally added interferent (Sigma-Aldrich), its final concentration was 50 µg∙mL^−1^ (50 ppm). For the experiments involving the commercial CE instrument, two sets of electrolytes were prepared covering a wide range of pH, 4–9, based on phosphate (NaH_2_PO_4_/Na_2_HPO_4_) and acetate (CH_3_COOH/CH_3_COONa) buffers (Sigma-Aldrich). It was decided to cross the buffering range in order to maintain the consistency of ionic composition throughout the tested pH range. The buffers were selected in such a way that their buffering capabilities complemented each other (acetate up to pH 6, phosphate over pH 6). Their ionic strength was 50 ± 10 mM. A different set of electrolytes was used in the case of portable CE setup (see Section 3.5). All other reagents were supplied by Avantor Performance Materials Poland. SA (Gliwice, Poland).

### 3.2. MST Instrumentation

The MST Monolith™ NT.115 instrument (NanoTemper Technologies GmbH, Munich, Germany) was used with a blue range fluorescence excitation (465–490 nm) and a 500–550 nm emission band pass filter. Standard capillaries dedicated to Monolith NT.115 were used (NanoTemper Technologies), their estimated internal diameter was around 500 µm. The excitation source (LED) power was 2%, 10% or 40%, as described in the above sections. The IR laser power was set to 50%. The system was thermostated at 22 °C. Measurements were carried out in triplicate and all parameter values were subsequently averaged for the further analysis.

### 3.3. CE Instrumentation

The PA800 plus CE instrument was used (Beckman-Coulter, Brea, CA, USA). The capillary temperature was 22 °C. The separation voltage was 30 kV. The additional forward pressure of 13.8 kPa (2.0 psi) was used to accelerate the analysis. The unmodified bare fused-silica capillary was used (Beckman-Coulter). Its total length was around 120 cm and it had a 75 µm internal diameter. The effective length to the LIF detector site was 20 cm. Between runs, the capillary was rinsed with 0.1 M NaOH for 1 min and background electrolyte for 2 min. Before the first use of the capillary on a working day, methanol for 10 min, 0.1 M HCl for 3 min, deionized water for 5 min, 0.1 M NaOH for 10 min and background electrolyte for 10 min were applied. For the fresh capillary conditioning, the latter sequence was used, but the duration of each individual step was doubled. The pressure applied equaled to 137.9 kPa (20 psi). Sample injection was conducted using the forward pressure of 6.9 kPa (1.0 psi) for 10 s. 

The CE system was equipped with the laser-induced fluorescence (LIF) detector operating at the 488 nm excitation wavelength and using the bandpass filter 520 ± 10 nm for the detection. 

### 3.4. CE-MST Interface

**Figure 4:**
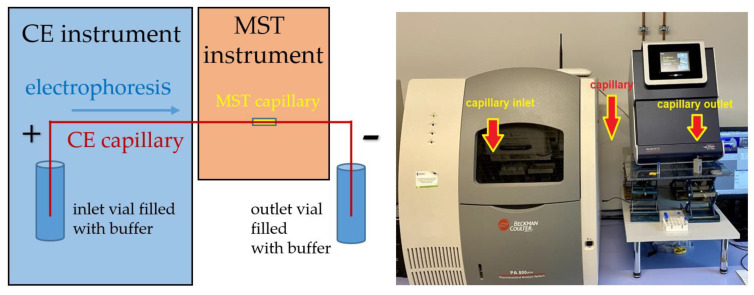
Schematic illustration of the CE-MST instrumentation (**left**) and its photograph (**right**).

The CE-MST interface consisted of inserting the narrower CE capillary directly into the broader MST capillary. The electrophoretic separation was stopped by switching off the voltage supply, which was followed by the MST measurement. It was attempted to be carried out exactly when the maximum of peak was observed via the MST detector. The detailed procedure was described in detail in our previous paper [18]. The visualization of the CE-MST coupling is shown in Figure 4 and Figures S1–S8 in the SM.

### 3.5. Home-Made Portable CE

The purpose-made instrument consisted of a positive high-voltage power supply (Spellman UM25P4, Spellman, Pulborough, UK) with two Pt/Ir wire electrodes (0.5 mm outer diameter, 5 cm long). The electrodes were inserted into two 1.5 mL conical vials (Eppendorf AG, Hamburg, Germany) with holes made in the lids, containing the background electrolyte. The system was coupled with the C^4^D detection system (eDAQ, Deninstone East, Australia), consisting of a C^4^D unit (ER225) and a capillary headstage (ET120). A fused silica capillary (Beckman-Coulter) of 100 cm total length and 75 µm internal diameter was used. Between runs, the capillary was rinsed with background electrolyte for 2 min. Before the first use of the capillary on a working day, methanol for 5 min, 1 M NaOH for 10 min, deionized water for 5 min and background electrolyte for 5 min were applied. For the fresh capillary conditioning, the same sequence was used. The sample injection was performed manually (siphoning) by elevating the injection capillary end to a height of 7 cm for 30 s. Separation was carried out at ambient temperature with a voltage of +11 kV applied at the injection side. The electrolytes prepared for measurements covered a pH range of 6–9 and consisted of L-histidine/2-(N-morpholino)ethanesulfonic acid (MES) buffer (pH 6) or L-arginine/MES buffer (pH 7–9). Their ionic strength was 10 ± 2 mM. All reagents for background electrolyte preparation were supplied by Sigma-Aldrich.

### 3.6. Methodology of pK_a_ Determination

The deprotonation of the phenol group of FITC was used as a model process because it causes a significant change in fluorescent properties, which are easy to detect and analyze [19]. The p*K*_a_ values obtained refer to the ionic strength of the particular electrolytes, and the recalculation to zero ionic strength was not performed, as it does not affect the comparison of MST with CE-MST.

#### 3.6.1. MST Method

The method for determining the acidity constant of FITC with the MST technique was described in detail in our recent article [19]. In brief, F_norm_ values (Equation (1)) were determined at different pH values above 6 to eliminate the undesirable influence of the carboxyl group on the obtained MST responses. To fit the F_norm_ values, a sigmoidal Boltzmann curve was used, see Equation (3):

(3) $$y=\frac{A-B}{1+e^{\left(x-x_{0}\right)/dx}}+B$$

where y is F_norm_, A and B are the model asymptotes standing for the non-ionized and ionized states, respectively, x_0_ is the inflection point standing for the p*K*_a_ (50% ionization), x is pH and dx is a constant of 0.434 determining the pH range in which the ionization degree increases.

#### 3.6.2. CE Electrophoretic Method

The acidity constant was determined using the same function, but different input data recorded during the same analytical run. The relationship between the relative electrophoretic mobility of FITC and pH was plotted for that purpose [20]. It was calculated based on the migration times recorded for FITC and AMAC, where the latter was used as a mobility standard which displayed constant mobility throughout the studied pH range, see Equation (4):

(4) $$\mu_{ep}=\beta \cdot \left(\frac{1}{t_{\mathrm{FITC}}}-\frac{1}{t_{\mathrm{AMAC}}}\right)$$

where µ_ep_ is the relative electrophoretic mobility (used as y in Equation (3) instead of F_norm_), β is a constant dependent on the capillary dimensions and applied electric voltage (insignificant with regard to p*K*_a_ value) and t_FITC_ and t_AMAC_ are migration times obtained for the FITC (analyte) and AMAC (mobility standard), respectively.

#### 3.6.3. CE Fluorescent Method

In this case, the dependence of FITC fluorescence on pH was utilized to determine acidity constant [19]. The FITC peak areas recorded by the LIF detector were divided by the peak areas recorded for AMAC in the same run, to eliminate the random error related to the injection step (A_FITC_/A_AMAC_ were used as y in the model described by Equation (3)). The fluorescence of AMAC was assumed to be constant over the studied pH range.

## 4. Conclusions

As evidenced by the presented results, the CE-MST technique allows us to determine the acid-base equilibrium constant in accordance with the standard MST approach. Integration of CE and MST is possible by means of a narrow and flexible CE capillary connecting the CE and MST instruments online, and by stopping the electrophoretic flow to enable thermophoretic analysis when the analyte zone reaches the MST detector. The presented method can be used to study acid-base properties of fluorophores, as well as other molecules upon their derivatization. It may be however tricky for small organic molecules. In the case of larger molecules, one should also remember about potential interference of multiple ionizable groups.

With regard to other potential applications, such a tandem technique can take a large advantage from performing electrophoretic separation of the sample directly before thermophoretic measurement, which can enable, e.g., the purification of the compound of interest from potential interferents. An interesting application might be chiral separation of isomeric drugs with CE, followed by their thermophoretic analysis focused on particular types of interactions. Secondly, CE-MST enables the recording of both thermophoretic and electrophoretic data with two independent detectors, providing a deeper insight into the interaction studied with additional orthogonal techniques. Finally, the use of CE may provide information about sample composition and its purity. Discovering these and other benefits requires further investigation. On the other hand, such an approach has some limitations, most importantly, is less efficient in terms of sensitivity, cost, time, user-friendliness and energy consumption. In addition, one should be aware that transferring the other known and new MST procedures to the CE-MST format may be far more problematic than for the quite simple method used herein. Therefore, CE-MST will probably never be as universal and versatile as MST, but rather dedicated to specific applications, difficult to handle with the standard approach.

The first results obtained with the portable CE system prove that the integration with the MST technique can be accomplished omitting the need to possess a large, expensive and energy-consuming CE instrument. These results seem especially promising for the future, since they show how to reconcile the pursuit for novel analytical capabilities with the idea of green chemistry [27,28] and practicality. Assessing how “white”, i.e., balanced in respect to different attributes, the CE-MST system is [29,30], will be the aim of our next work.

## Figures and Tables

**Table 1:** Fluorescence intensity (relative units) of FITC measured by the MST detector without switching on the IR laser, in a phosphate-based electrolyte of different pH values, using the MST and CE capillaries.

pH	MST Capillary (wide)			CE Capillary (narrow)	NSR
	LED 2%, C_FITC_ 50 ng∙mL^−1^	LED 10%, C_FITC_ 50 ng∙mL^−1^	LED 40%, C_FITC_ 50 ng∙mL^−1^	LED 10%, C_FITC_ 1 µg∙mL^−1^	
4.03	0.89	10.62	49.13	24.19	8.78
4.50	1.10	13.25	61.80	43.18	6.14
5.00	1.14	14.39	67.63	53.62	5.37
5.50	1.28	16.30	76.00	70.47	4.63
6.00	2.29	30.97	142.73	96.60	6.41
6.50	4.71	65.67	298.17	138.87	9.46
7.00	10.17	143.00	642.67	172.50	16.58
7.50	14.83	212.17	948.33	208.83	20.32
8.00	17.23	247.33	1106.67	200.83	24.63
9.00	20.07	286.83	1281.67	199.57	28.75
Mean	7.37	104.05	467.48	120.87	13.11

LED – relative power of light emitting diode (intensity of excitation light set up with the MST device); NSR – normalized signal ratio (it means how many times the sensitivity in MST capillary is higher than in the CE capillary, Equation (2)).

## Data Availability

Raw data supporting the manuscript content are publicly available using the link: (https://ruj.uj.edu.pl/xmlui/handle/item/297982; DOI:10.26106/rzg7-x025) (accessed on 3 August 2022).

## Supplementary Materials

The following supporting information can be downloaded at: https://www.mdpi.com/article/10.3390/molecules27155010/s1: Table S1. Fluorescence intensity (relative units) of FITC measured by the MST detector before switching on the IR laser, in the acetate-based electrolytes of different pH values, using the MST and CE capillaries, Table S2. Percentage relative standard deviation (RSD%, n = 3) of Fnorm values obtained for the phosphate-based electrolytes and 5 s of heating, Table S3. Percentage relative standard deviation (RSD%, n = 3) of F_norm_ values obtained for the phosphate-based electrolytes and 30 s of heating, Table S4. Percentage relative standard deviation (RSD%, n=3) of F_norm_ values obtained for the acetate-based electrolytes and 5 s of heating, Table S5. Percentage relative standard deviation (RSD%, n = 3) of F_norm_ values obtained for the acetate-based electrolytes and 30 s of heating, Figure S1. The MST device equipped with the CE capillary handled by a syringe. This preliminary setup was used to check out the capability of inserting the CE capillary directly inside the MST capillary, to realize the CE-MST interface. In this setup the sample was introduced from the vial (on the left) using vacuum generated by the syringe (on the right), Figure S2. The CE capillary installed directly inside the broader MST capillary, mounted and immobilized on the conventional tray used for MST measurements, Figure S3. The syringe connected hermetically with the CE capillary, used in the setup presented in Fig.S1, Figure S4. The CE and MST devices connected on-line by the single CE capillary (the capillary is hardly visible due to its small dimensions), Figure S5. The outlet part of the CE capillary immersed in a vial containing background electrolyte, and the platinum electrode used to close the electric circuit and enable electrophoretic separation, Figure S6. The CE capillary inserted inside the MST capillary, fixed on the tray. To enable signal detection, a light transparent window was burned off into the CE capillary, of ca. 2-3 mm length. After the CE capillary was immobilized, the window was located exactly in the center of the MST tray, Figure S7. The position of the inlet and outlet of the CE capillary used in the CE-MST technique. To avoid straining the CE capillary that may cause relocation of the detection window site in the MST device, an excess portion of the capillary of about 15 cm long was kept outside the instruments, Figure S8. The home-made portable CE setup integrated with the MST device.
