# Supplementary material for: The First Online Capillary Electrophoresis-Microscale Thermophoresis (CE-MST) Method for the Analysis of Dynamic Equilibria—The Determination of the Acidity Constant of Fluorescein Isothiocyanate
Source: Molecules. 2022 Aug 6;27(15):5010. doi: 10.3390/molecules27155010 (PMC9370695; doi:10.3390/molecules27155010)
Supplement: Supplementary file 1 [file molecules-27-05010-s001.zip › molecules-1837683-supplementary.pdf]

Supplementary Material (SM)

The First Online Capillary Electrophoresis-Microscale Thermophoresis (CE-MST) Method for the Analysis of Dynamic Equilibria—The Determination of the Acidity Constant of Fluorescein Isothiocyanate

Paweł Mateusz Nowak*, Maria Klag, Gabriela Kózka, Małgorzata Gołąb, Michał Woźniakiewicz

Jagiellonian University in Kraków, Faculty of Chemistry, Department of Analytical Chemistry, Gronostajowa St. 2, 30-387 Kraków, Poland

Corresponding author: dr Paweł Mateusz Nowak, Gronostajowa St. 2, 30-387 Kraków, Poland, tel./fax +48 12 686 2409, e-mail: pm.nowak@uj.edu.pl

Additional experimental data

Table S1. Fluorescence intensity (relative units) of FITC measured by the MST detector before switching on the IR laser, in the acetate-based electrolytes of different pH values, using the MST and CE capillaries.

	MST capillary			CE capillary	
**pH**	**LED 2%, C_{FITC} 50 ng·mL^{-1}**	**LED 10%, C_{FITC} 50 ng·mL^{-1}**	**LED 40%, C_{FITC} 50 ng·mL^{-1}**	**LED 10%, C_{FITC} 1 μg·mL^{-1}**	**NSR**
4.05	1.63	21.07	96.33	30.80	13.68
4.54	1.35	17.47	81.23	38.15	9.16
5.04	1.43	18.71	87.67	49.73	7.53
5.54	1.87	24.76	111.23	65.47	7.56
6.05	2.72	37.92	174.77	100.00	7.58
6.55	5.19	72.43	329.00	143.27	10.11
7.05	10.07	143.07	643.00	174.90	16.36
7.51	15.06	214.33	960.67	198.73	21.57
8.06	19.30	275.83	1236.00	214.50	25.72
9.07	16.84	240.50	1082.00	213.00	22.58
Mean	7.55	106.61	480.19	122.86	14.19

LED – Relative power of light emitting diode (intensity of excitation light set up with the MST device); NSR – normalized signal ratio (the value obtained for the MST capillary with LED set up at 10% divided by the values obtained for the CE capillary and the same LED, normalized in respect to the difference in FITC concentrations; it means how many times the sensitivity in MST capillary is higher than in the CE capillary)

Table S2. Percentage relative standard deviation (RSD%, n=3) of F_{norm} values obtained for the phosphate-based electrolytes and 5 seconds of heating.

pH	MST capillary			CE capillary
	**LED 2%, C_{FITC} 50 ng·mL^{-1}**	**LED 10%, C_{FITC} 50 ng·mL^{-1}**	**LED 40%, C_{FITC} 50 ng·mL^{-1}**	**LED 10%, C_{FITC} 1 µg·mL^{-1}**
4.03	1.85	0.87	0.04	0.45
4.50	0.91	0.28	0.28	0.46
5.00	1.01	0.16	0.14	0.15
5.50	1.62	0.48	0.04	0.19
6.00	1.57	0.22	0.10	0.19
6.50	0.21	0.29	0.12	0.05
7.00	0.64	0.15	0.10	0.12
7.50	0.10	0.16	0.06	1.75
8.00	0.35	0.02	0.08	0.06
9.00	0.20	0.10	0.13	0.06
Mean	0.85	0.27	0.11	0.35

LED – Relative power of light emitting diode (intensity of excitation light set up with the MST device)

Table S3. Percentage relative standard deviation (RSD%, n=3) of F_{norm} values obtained for the phosphate-based electrolytes and 30 seconds of heating.

pH	MST capillary			CE capillary
	**LED 2%, C_{FITC} 50 ng·mL^{-1}**	**LED 10%, C_{FITC} 50 ng·mL^{-1}**	**LED 40%, C_{FITC} 50 ng·mL^{-1}**	**LED 10%, C_{FITC} 1 µg·mL^{-1}**
4.03	1.44	0.03	0.30	0.11
4.50	3.00	0.38	0.40	0.37
5.00	1.51	0.41	0.14	0.40
5.50	1.01	0.46	0.05	0.06
6.00	0.95	0.10	0.21	0.04
6.50	0.75	0.02	0.43	0.20
7.00	0.46	0.17	0.68	0.57
7.50	0.72	1.37	0.62	0.25
8.00	0.02	0.19	1.04	0.10
9.00	0.09	0.23	0.80	0.23
Mean	0.99	0.34	0.47	0.23

LED – Relative power of light emitting diode (intensity of excitation light set up with the MST device)

Table S4. Percentage relative standard deviation (RSD%, n=3) of F_{norm} values obtained for the acetate-based electrolytes and 5 seconds of heating.

pH	MST capillary			CE capillary
	**LED 2%, C_{FITC} 50 ng·mL^{-1}**	**LED 10%, C_{FITC} 50 ng·mL^{-1}**	**LED 40%, C_{FITC} 50 ng·mL^{-1}**	**LED 10%, C_{FITC} 1 µg·mL^{-1}**
4.05	1.65	0.16	0.18	0.34
4.54	0.83	0.06	0.10	0.46
5.04	3.15	0.47	0.12	0.22
5.54	0.85	0.22	0.28	0.28
6.05	0.98	0.18	0.10	0.10
6.55	0.22	0.22	0.21	0.11
7.05	0.28	0.19	0.02	0.09
7.51	0.36	0.11	0.14	0.17
8.06	0.18	0.01	0.11	0.11
9.07	0.62	0.20	0.11	0.05
Mean	0.91	0.18	0.14	0.19

LED – Relative power of light emitting diode (intensity of excitation light set up with the MST device)

Table S5. Percentage relative standard deviation (RSD%, n=3) of F_{norm} values obtained for the acetate-based electrolytes and 30 seconds of heating.

pH	MST capillary			CE capillary
	**LED 2%, C_{FITC} 50 ng·mL^{-1}**	**LED 10%, C_{FITC} 50 ng·mL^{-1}**	**LED 40%, C_{FITC} 50 ng·mL^{-1}**	**LED 10%, C_{FITC} 1 µg·mL^{-1}**
4.05	0.72	0.48	1.44	0.52
4.54	0.43	0.19	1.63	0.34
5.04	1.15	1.12	0.90	0.11
5.54	1.37	0.08	0.53	0.17
6.05	0.56	0.21	0.21	0.29
6.55	0.72	0.12	0.44	0.36
7.05	0.30	0.33	0.66	0.13
7.51	0.64	0.45	0.27	0.69
8.06	0.41	0.18	0.58	0.21
9.07	0.27	0.07	0.97	0.25
Mean	0.66	0.32	0.76	0.31

LED – Relative power of light emitting diode (intensity of excitation light set up with the MST device)

Visualization of the preliminary setup

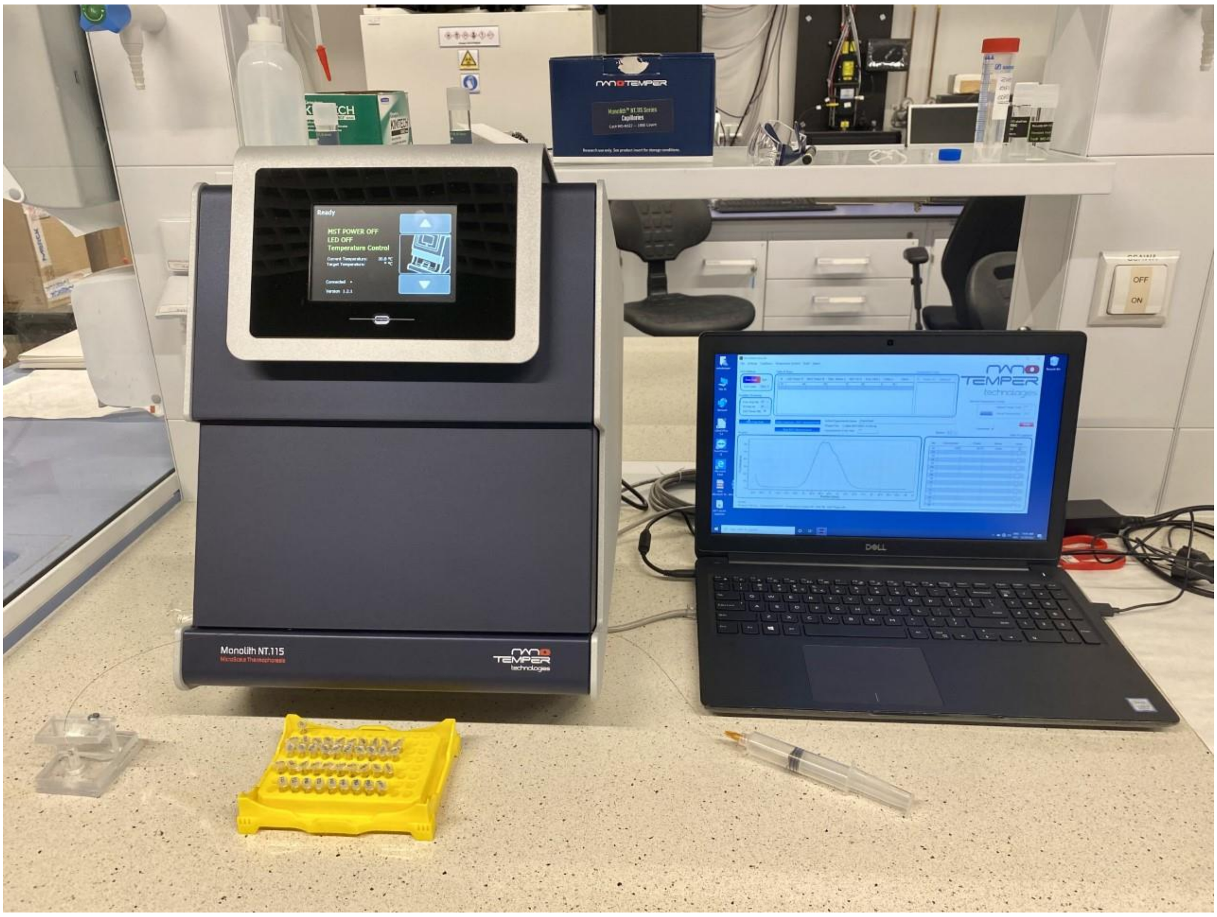

Figure S1. The MST device equipped with the CE capillary handled by a syringe. This preliminary setup was used to check out the capability of inserting the CE capillary directly inside the MST capillary, to realize the CE-MST interface. In this setup the sample was introduced from the vial (on the left) using vacuum generated by the syringe (on the right).

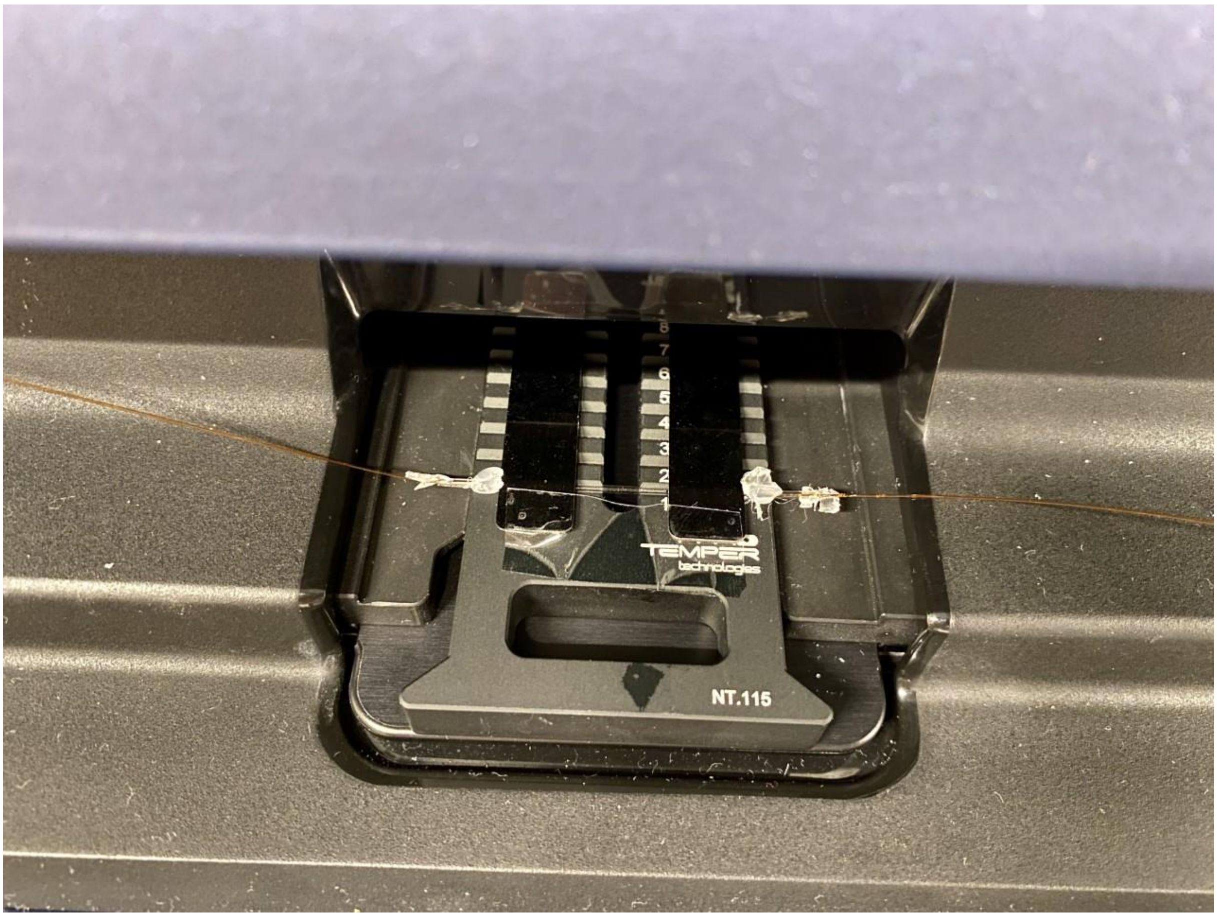

Figure S2. The CE capillary installed directly inside the broader MST capillary, mounted and immobilized on the conventional tray used for MST measurements.

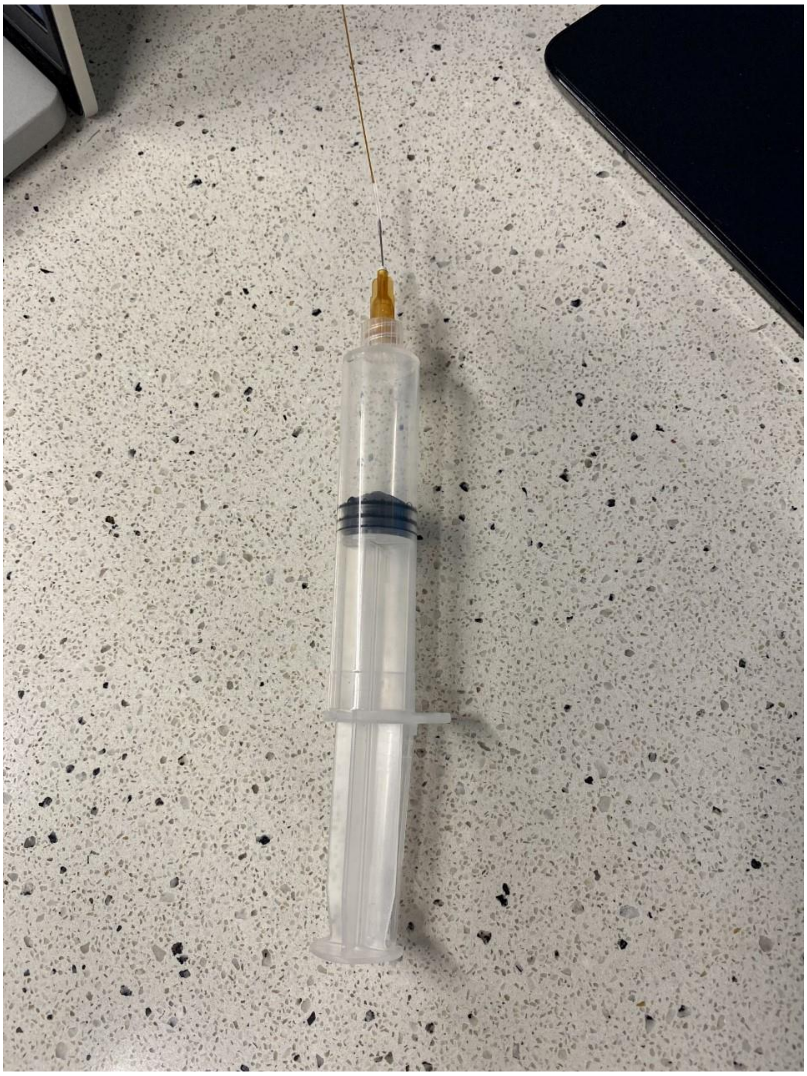

Figure S3. The syringe connected hermetically with the CE capillary, used in the setup presented in Fig.S1.

Visualization of the target CE-MST coupling

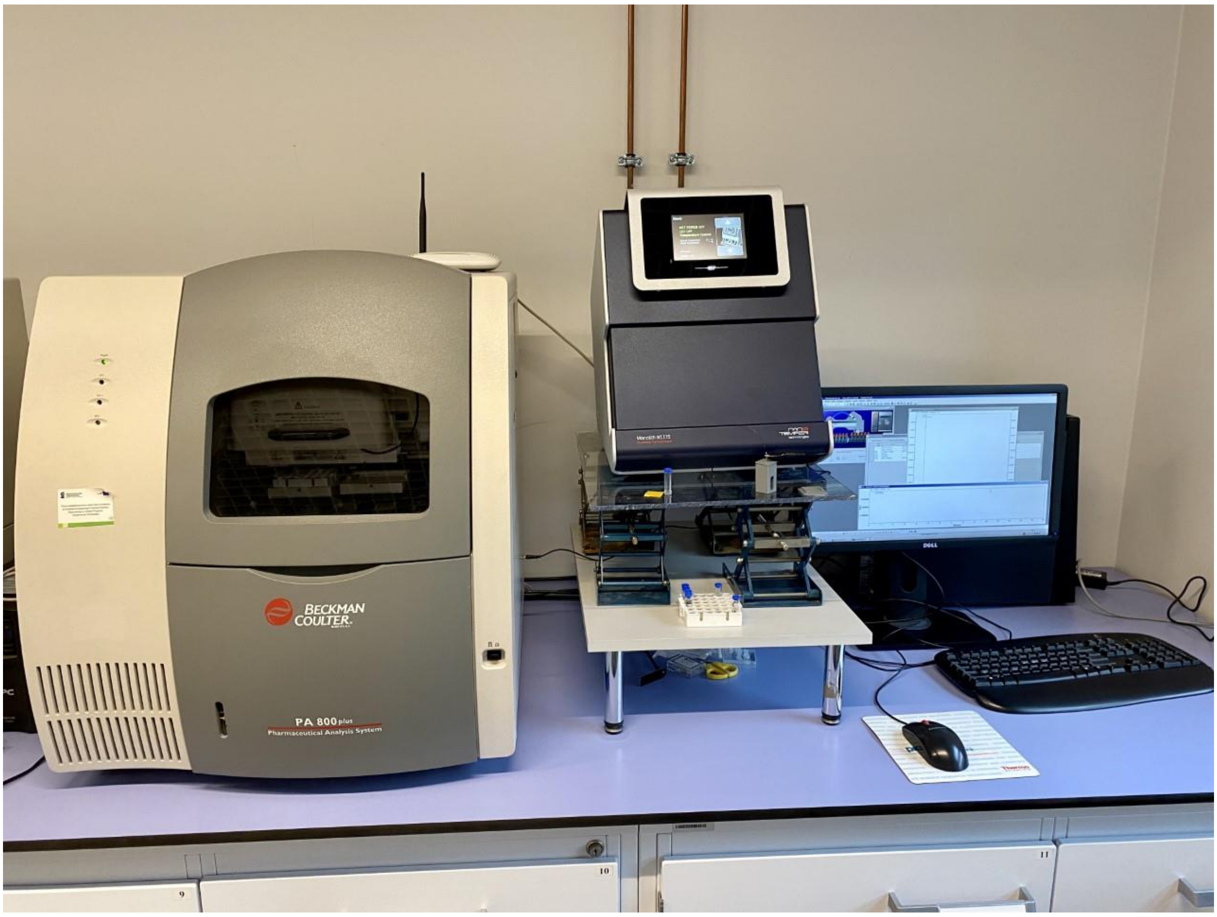

Figure S4. The CE and MST devices connected on-line by the single CE capillary (the capillary is hardly visible due to its small dimensions).

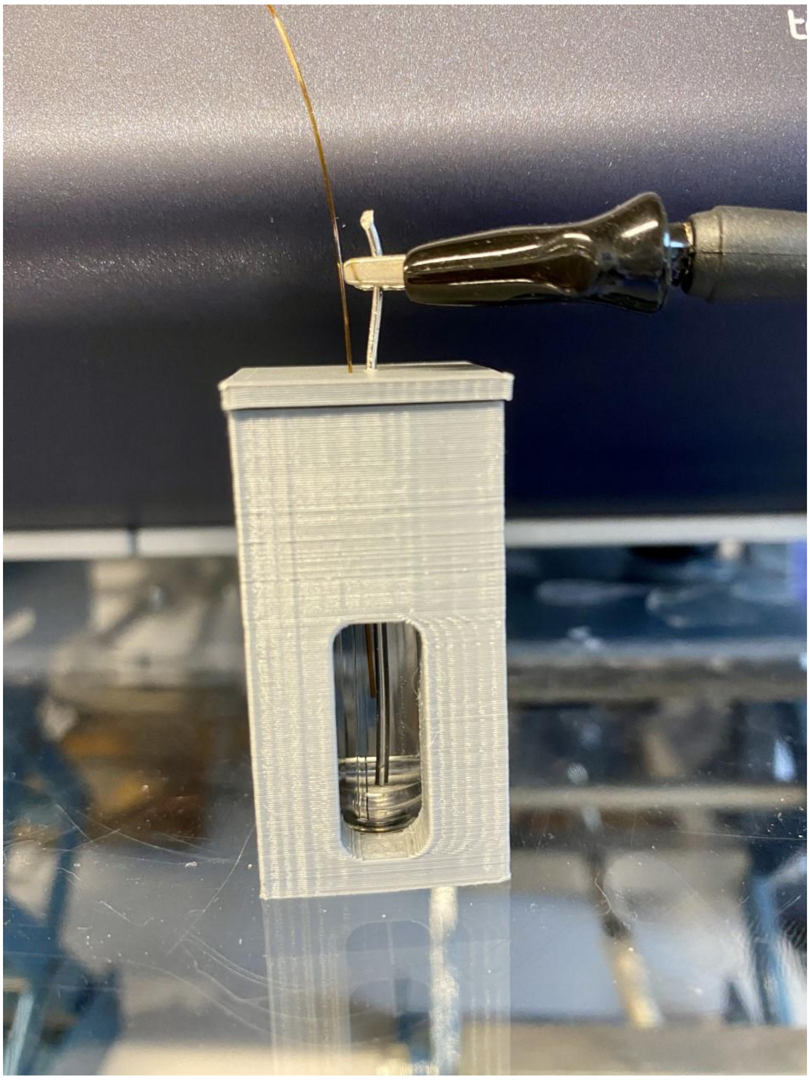

Figure S5. The outlet part of the CE capillary immersed in a vial containing background electrolyte, and the platinum electrode used to close the electric circuit and enable electrophoretic separation.

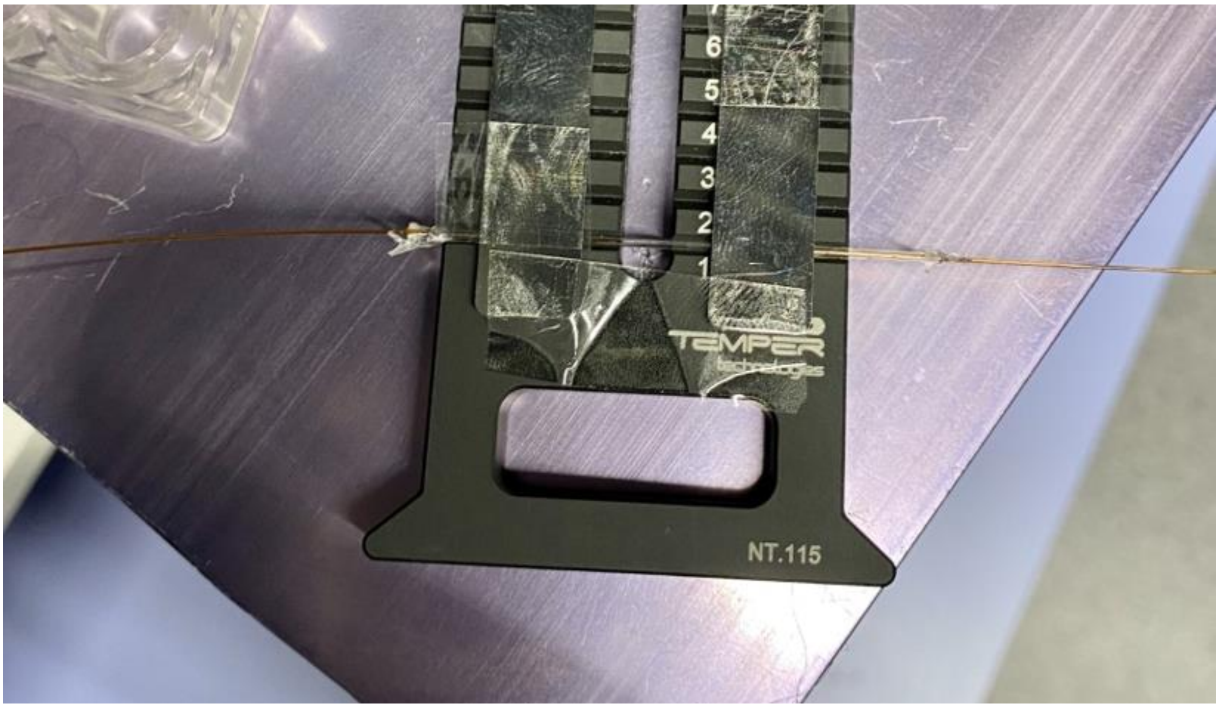

Figure S6. The CE capillary inserted inside the MST capillary, fixed on the tray. To enable signal detection, a light transparent window was burned off into the CE capillary, of ca. 2-3 mm length. After the CE capillary was immobilized, the window was located exactly in the center of the MST tray.

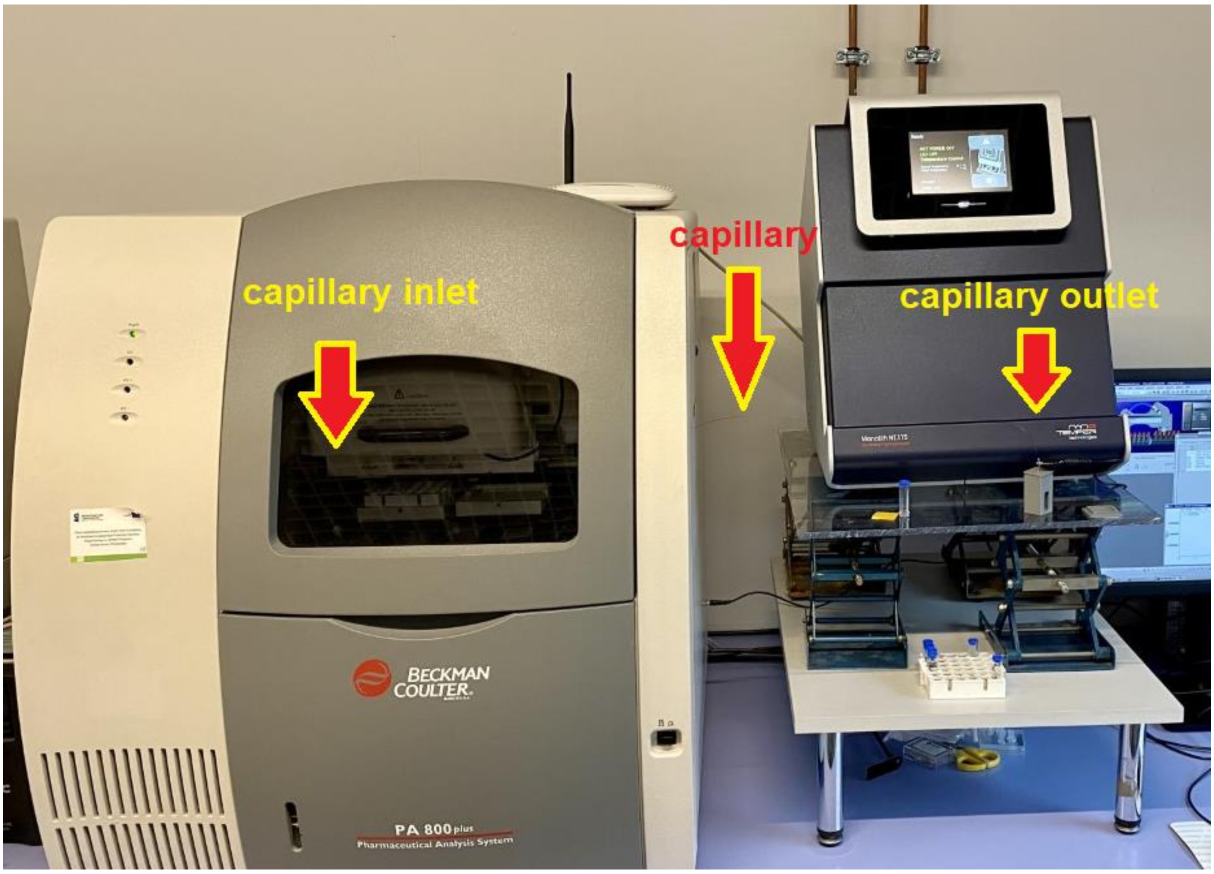

Figure S7. The position of the inlet and outlet of the CE capillary used in the CE-MST technique. To avoid straining the CE capillary that may cause relocation of the detection window site in the MST device, an excess portion of the capillary of about 15 cm long was kept outside the instruments.

Visualization of the alternative low-cost CE-MST coupling

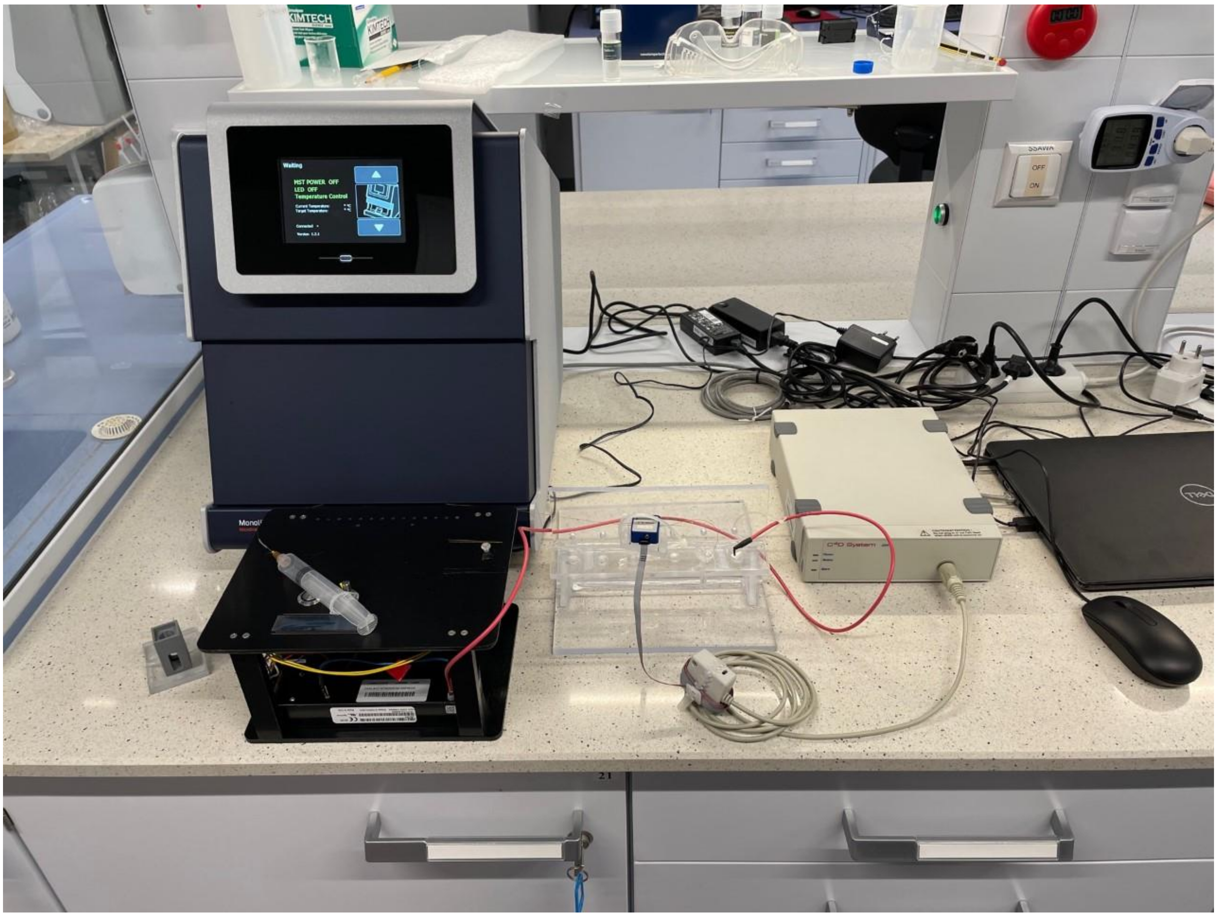

Figure S8. The home-made portable CE setup integrated with the MST device.
